# Eye Movements in Mild Traumatic Brain Injury: Ocular Biomarkers

**DOI:** 10.16910/jemr.15.2.4

**Published:** 2022-06-16

**Authors:** Matthew A. McDonald, Samantha J. Holdsworth, Helen V. Danesh-Meyer

**Affiliations:** Department of Ophthalmology, University of Auckland, New Zealand; Mātai Medical Research Institute, Gisborne, New Zealand; Department of Anatomy and Medical Imaging, University of Auckland, New Zealand; Eye Institute, Auckland, New Zealand

**Keywords:** concussion, sport-related concussion, mild traumatic brain injury, mTBI diagnosis, mTBI epidemiology, mTBI pathophysiology

## Abstract

Mild traumatic brain injury (mTBI, or concussion), results from direct and indirect trauma to the head (i.e. a closed injury of transmitted forces), with or without loss of consciousness. The current method of diagnosis is largely based on symptom assessment and clinical history. There is an urgent need to identify an objective biomarker which can not only detect injury, but inform prognosis and recovery. Ocular motor impairment is argued to be ubiquitous across mTBI subtypes and may serve as a valuable clinical biomarker with the recent advent of more affordable and portable eye tracking technology. Many groups have positively correlated the degree of ocular motor impairment to symptom severity with a minority attempting to validate these findings with diffusion tract imaging and functional MRI. However, numerous methodological issues limit the interpretation of results, preventing any singular ocular biomarker from prevailing. This review will comprehensively describe the anatomical susceptibility, clinical measurement, and current eye tracking literature surrounding saccades, smooth pursuit, vestibulo-ocular reflex, vergence, pupillary light reflex, and accommodation in mTBI.

## Introduction

Mild traumatic brain injury (mTBI, also known as concussion) is characterised by the World Health Organization (WHO) as a neurobehavioural phenomenon caused by external physical forces (i.e. trauma) with no penetrating head injury (32). This has major health significance with mTBI recognized as a leading cause of morbidity, resulting in significant health and economic consequences. In fact, half the world’s population is expected to experience a form of head injury during their lifetime (107). Each year, 50 million people suffer from mTBI (at least 6 per 1,000 globally) and persistent symptoms are common (79; 107). Currently there are no rapidly available biomarkers to indicate when the brain has suffered an mTBI or recovered. An objective biomarker could be used to guide medical decisions to mitigate the effects of repeated mTBI, particularly relevant to contact sports players. Evidence has demonstrated that eye-tracking abnormalities are present in patients with mTBI due to the complex integration of multiple brain networks required for cognition and ocular motor control. Following “Eye Movements in Mild Traumatic Brain Injury: Clinical Challanges”, this review describes potential ocular biomarkers to encourage objective assessments in these patients: saccades, smooth pursuit, vestibulo-ocular reflex, vergence, pupillary light response, and accommodation.

## Methods of Literature Search

The MEDLINE and PubMed databases were used for this review. Searched key words were chosen appropriately to for each section of this article. For example, keywords related to “Saccades” used to search each database were saccades, eye movements, eye tracking, pupil-tracking, mild traumatic brain injury, oculomotor, ocular motor, mTBI, concussion, sport-related concussion, postconcussion syndrome, latency, velocity, gain, biomarker, along with combinations of pertinent Boolean operators. We included studies related to each section and excluded any qualitative studies, in addition to non-academic journal articles (e.g. newsletters/ magazines) and case reports. We screened the reference list from each included study to find additional articles in this area. Non-English articles were not found in this area and therefore not included.

## Ocular Biomarkers in mTBI

Biologic markers, termed ‘biomarkers’, are classified by The Biomarkers Definition Working Group as “a characteristic that is objectively measured and evaluated as an indicator of normal biological processes, pathogenic processes, or pharmacologic responses to a therapeutic intervention” (67). In mTBI, a number of areas require such a marker: the diagnosis of acute mTBI (positive and negative predicative value with index of severity), post-concussion syndrome (to exclude other confounding illness), and recovery (resolution of the abnormal ‘biomarker’).

The visual system contains widely distributed networks which may be vulnerable to pathophysiologic changes after mTBI (170). A complex system of white matter tracts control eye movements, from the frontal lobe (cingulum and inferior fronto-occipital fasciculus) to the brain stem (medial longitudinal fasciculus, medial lemniscus, spinothalamic tract, central tegmental tract, and cerebellar peduncles). Cognition and attention (cortical gray matter regions) are highly integrated into these pathways. As a result, ocular motor testing with more complex tasks (e.g. anti-saccades) may serve as a more sensitive marker of brain injury.

In a controlled environment using eye tracking technology, it has been shown that pupillary responses, smooth pursuit (following a target slowly with the eyes), saccades (looking from left to right), conjugacy (how the eyes work together as a pair), and anti-saccades (looking in the opposite direction of where a target appears) are affected to varying degrees. More recently, portable eye-tracking devices have advanced to such an extent that researchers are able to evaluate these movements in a more practical clinical setting.

### Saccades

Saccades are defined as rapid eye movements between two points. Although these have an automated, reflexive component through the superior colliculus’ signals from the retina and subcortical structures (127; 154), they are not immune to cognitive and attention effects (37; 135; 153). Numerous cortical regions, such as the partietal cortex, frontal eye fields, supplementary eye fields, and dorsolateral prefrontal cortex (DLPFC) influence this reflex, particularly with anti-saccades (gaze directed in the opposite direction of the stimulus) (28) and memory-guided saccades (saccades performed to remembered target location no longer on display) (48).

The reliance on multiple cortical areas which communicate via white matter tracts make these movements vulnerable to mTBI. For example, the corpus callosum and superior colliculus, instrumental in saccade function, have been shown to be at risk of diffuse axonal injury in mTBI (33; 80; 176).

### Clinical Measurement

**Figure 1.**
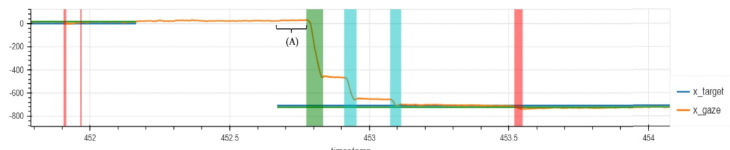
Horizontal saccade task on an eye tracking device operating at 200 Hz. The red bars identify microsaccade events. The vertical green bar highlights a saccade toward the target with blue bars identifying corrective saccades. (A) represents saccade latency (reaction time from stimulus presentation to initiation of saccade). The blue and green horizontal lines represent the target location while the orange line represents the healthy participant’s gaze trajectory. X-axis represents time in seconds while the Y-axis represents distance in pixels. Original data acquired using a 200 Hz eye tracking device on a healthy 42-year-old male volunteer.

Clinical evaluation of saccades in mTBI patients will only reveal gross abnormalities observable to the naked eye. These are tested through the examiner holding out two fingers, approximately one meter apart. The patient is asked to look from one finger to the next (30 degrees each way) as quickly as possible for 10 repetitions. The examiner may notice saccade dysmetria which terms the over- or under-shoot of the eye on target and is accompanied by corrective saccades. This is repeated for vertical saccades and patients may be assessed for headache, vertigo, nausea, and ‘fogginess’ as part of the Vestibular/ Ocular Motor Screening Tool (VOMS) (129). The Developmental Eye Movement Test (DEM) is a paper-based test which instructs a participant to read equally spaced numbers, both horizontally and vertically, incorporating attention and language. There is no correlation between saccadic eye movement skills or symptomology when compared to eye tracking measures (12). The King-Devick test is another office-based saccade task where a participant reads rows of randomly assorted and spaced numbers (57). However, both the VOMS and DEM tasks lack objectivity and speficity as they show poor correlation to more accurate, quantitative measures found on eye tracking (Figure 1) (12). Ayton and colleagues found no correlation between saccadic eye movement skills or symptomology when comparing the DEM to eye tracking measures. The DEM was only correlated to reading performance and visual processing speed (12).

### Saccades and mTBI

Saccadic eye movements are a common outcome measure in eye tracking studies due to their relative ease of measurement as well-defined events. Eye tracking studies on mTBI participants have revealed abnormal saccade latencies (delayed initiation of saccades as a marker of reaction time) and accuracy values (amplitude and gain for the eye to meet the correct target position) which are detailed below in ‘Reflexive Saccade Tasks’. More complex saccade-based tasks (e.g. memory-guided or anti-saccades) involve a higher cognitive load which may show greater sensitivity in eliciting abnormalities.

### Reflexive Saccade Tasks

Multiple groups have quanitifed saccadic abnormalities using eye tracking in mTBI patients. Cochrane and colleagues evaluated 28 sport-related mTBIs (within 2 weeks of injury; 87 controls) in a reflexive saccadic task which revealed decreased accuracy of both horizontal and vertical saccades, in addition to increased latencies relative to control (38). This was the only study which evaluated test re-test reliability (measured in their control cohort) and was found to be generally poor for both accuracy and smooth pursuits, except for saccade latencies. Their eye tracker operated at 100 Hz and analysis was not detailed (authors report the use of commercial software).

Danna-Dos-Santos et al. studied a cohort 36 mixed-cause mTBI patients averaging 43 months post-injury (+/- 52 months) which showed only lower accuracy in the first initial phase of a reflexive saccades, in addition to increased latency (46). There was no difference between groups for overall saccadic accuracy which was considered to be due to effective corrective saccades in the mTBI cohort, but these were not measured or quanitifed. This group used the same eye tracking hardware as Cochrane and colleagues and did not detail calibration or analysis, making it difficult to draw conclusions.

DiCesare and colleagues (acute cohort of 17 acute mTBI patients with an average of 7.6 days post-injury) also reported higher saccade latency in addition to increased fixation error on targets in between saccades (49). Velocities were not significantly different, nor number of self-paced saccades. However, their eye tracker operated at only 60Hz and analysis is not detailed (authors report “custom Matlab scripts”). Their data for each eye was averaged together and smoothed (moving average filter every five samples, 83 ms) with interpolation to remove gaps, which may have buried significant indices further.

Hunfalvy and colleagues report a novel measure in a cohort of 64 mTBIs (<30 days post-injury; 51 age-matched controls): saccade amplitude to velocity ratio. There were two further groups of moderate (n=64) and severe (n=23) TBIs. There was no main differences between these subgroups, except for larger variability in the severe TBI cohort. Reflexive saccades proved significant for both horizontal (sensitivity of 0.77, specificity 0.78) and vertical saccades (sensitivity of 0.64, specificity 0.65), although this relationship could have been explored further with correlation to symptom severity and follow-up with recovery (82). In addition, their use of their Right Eye platform, a financial interest, functioned at only 120 Hz and analysis was not detailed, nor their calibration or test-retest reliability, making it difficult to interpret and replicate.

### Complex Saccade Tasks

Self-paced saccades (asking a patient look left and right as many times as possible within a pre-defined time limit) was reduced in select studies of mTBI patients. Taghdiri and colleagues evaluated 59 participants 26 months post-injury (+/- 63 months) who were pesistently symptomatic, and two patients one-month post-injury. Over a 30-second period, these patients made 45-75 self-paced saccades, whereas unaffected patients made 74-84 of these movements. This was correlated to disruption of the left uncinate fasciculus and left cingulum on diffusion MRI (DTI; diffusion tensor imaging) (170). Given the wide range of months post-injury and lack of subgroup analysis, it is difficult to infer the generalizability of these results. However, Heitger and colleagues also revealed a reduction in self-paced saccades between mTBI (<10 days post-injury) and controls, albeit without neuroimaging (73). Reflexive saccade measures did not show any difference between the two groups.

Johnson and colleagues reported decreased self-paced saccades with higher positional errors and increased latencies, in addition to impaired amplitudes during anti-saccade and memory-guided saccade tasks in a small cohort of 9 athletes within 7 days of mTBI (88). Velocities and latencies on reflexive saccades were not significant which was likely too inaccurate to evaluate due to their low frequency eye tracking at 60 Hz. Their findings correlated to higher levels of brain activation across multiple regions on fMRI, speculated to occur from increased brain ‘effort’ (recruiting brain regions not typically involved in this task). Their follow up study on 7 of these patients showed improvement in self-paced saccades, memory-guided saccades, and anti-saccades between the acute-phase of injury and follow up (30 days) (87). However, at 30-days, they were still unable to reach the standard of their healthy counterparts which was also evident on functional MRI (fMRI), showing increased areas of activation (87). This small study used a 60Hz eye tracker with commercial and custom data analysis with limited detail. Additionally, test-retest reliability of their fMRI indices were not reported.

A further imaging study by Tyler and colleagues revealed impaired latencies (prolonged) and slower peak velocities in their cohort of 12 mTBI patients (0.2 to 36 years post-mTBI). These patients showed a blunted response in the abducens nuclei and supra-oculomotor area on fMRI (181). However, these results must be interpreted with caution due to their heterogenous and underpowered sample size. There was only a one-minute recording session with 12 repeats per eye movement. Altogether, the chance of observing a significant result due to chance alone is high.

Balaban’s group assessed 100 acute mTBIs (within 4 days of injury with follow ups at one- and two-weeks post-injury) and 200 age- and gender-similar controls with a 100Hz binocular eye tracker (commercial software) (16). Their group found significantly impaired prosaccade error rate (inhibition of erroneous saccades during anti-saccade task) and altered predictive saccade performance (saccade errors in response to predictable stimuli). Their test battery (which also included head-impulse testing for VOR and reaction times), yielded a diagnostic efficacy of 89% sensitivity and 95% specificity on their data. Their follow up study (with an additional 6 mTBIs and 100 further controls) reported outcomes at 7-10 and 14-17 days post-injury, revealing similar findings: predictive saccade response and pro-saccade error rate (anti-saccades) differentiated mTBI from controls, in addition to corrrelating with recovery (78). Their added statistical measure of ‘mean area under the main sequence curve’ for horizontal saccades also proved significant with ongoing dysfunction at 2 weeks. However, it is worth noting their control group only underwent one test and the test-retest reliability of these measures were not assessed.

Kelly’s group performed a similar test battery using the same eye tracker (Neuro Kinetics, a financial interest) in 50 high-school sport-related mTBIs (40 patients <22 days post-injury with 10 patients 27-328 days, mean 22.1; 170 athletic controls) and revealed decreased saccade velocities only when combined with the task of pressing a buzzer at the same time, increasing the cognitive load (91). Reflexive saccade measures were not affected. Their wide range of post-injury timepoints may have skewed data with recovery effects, but their use of a combined task shows promise for increasing the sesnsitivity of these measures.

In a cohort of 71 military personnel (75 age-similar controls) suffering from ongoing symptom burden post-mTBI (3 months to 5 years post-injury without neuroimaging), Wetzel’s group increased participants’ cognitive load through the evaluation of saccades during a reading task (186). They reported reduced saccadic amplitudes (particularly forward saccadic amplitudes) with velocities were unaffected. Therefore, increasing brain ‘effort’ (cognitive load) may produce more subtle abnormalities in those with some degree of recovery post-injury, as suggested previously. This would further provide support for a cognition-effect on the saccadic reflexes.

A dynamic evaluation of saccadic eye movements was performed by Murray and colleagues on the Wii Balance Board© with a virtual soccer heading game using a 240Hz monocular eye tracker (131). The mTBI cohort of 18 sport-related concussions (24-48 hours post-injury, with 18 athletic control participants) expanded on previous work (130). Although saccade count was not different between groups, their group revealed increased saccade amplitude, velocity, and decreased smooth pursuit velocity (target lag was increased, although this was not measured directly as the stimulus target speed was not known). This was considered to occur as a result of poorly integrated spatial and motion data with the mTBI group, requiring more catch-up saccades to reduce retinal slip (131). Heitger and collagues’ earlier 2002 study of 30 mixed-cause mTBIs within 9 days post-injury (30 controls age-, gender- and education-matched) arrived at a similar conclusion, reporting a series of directional errors in a 3-step saccade sequence (large saccade gain and position errors) which also suggested the diminished spatial accuracy in these patients (72). It is worth noting this group included a more severe spectrum of mTBI with 25 patients experiencing loss of conciousness and post-traumatic amnesia of 3 minutes to 4 hours which may have increased their sensitivity of detection.

Other complex tasks, such as anti-saccades, have been associated with increased symptom burden for mTBI patients with correlation to white matter disruption (176). Ting and colleagues’ studied a cohort of 11 acute mTBI and 15 with persistent symptoms (median of 8 months post-injury) and only found anti-saccade latency to be a useful measure (correct anti-saccades, duration, amplitude, and velocity were not significant between all groups). Their increased latency correlated to disrupted diffusion MRI measures of the splenium of the corpus callosum (acute cohort) and the corticospinal tract (persistent symptom cohort). In this small study, 7 patients in the acute cohort required visual correction and were asked to wear contact lenses with the proportion who followed this advice not reported. Additionally, details on the eye tracker frequency and precision were not reported. Commercial software was used for analysis and not detailed further, limiting the wider application of these results.

Limited studies have shown anti-saccade errors following mTBI, most notably in latency and error rate (74; 75; 77). Phillipou and colleagues’ paediatric cohort (mean age 13; 26 mTBI and 29 age-matched controls) showed mixed results: once children with multiple previous mTBI were excluded, mTBI patients made fewer anti-saccade errors acutely, but when they did, it took longer to correct. Increased anti-saccade and prosaccade latency was only apparent at the third time point (6 months). Patients with multiple previous mTBIs (n=7) only showed a group difference in correctional saccade latency at 3 months. The authors inferred that their head injury affected their brain development as the comparison group improved through repeat testing (possible practice bias). There was also no difference in self-paced saccades in the acute mTBI cohort vs control, contrary to adult cohorts (142). Their group hypothesized that the reduced sensitivity to the target appearing during the anti-saccade task made it paradoxically easier to inhibit the reflexive saccade, increasing their accuracy. Another theory centred around an increase in extracellular serotonin levels in the acute phase of mTBI which may have postively influenced the inhibitory pathways involved in anti-saccade suppression. This should be considered as a tenuous conclusion given their small sample size and potential for multiple comparison bias.

A study using artificial neural networks (ANN) analyzed anti-saccade parameters in a group of 32 mTBI patients, 25 post-concussion syndrome (PCS) patients (ongoing symptoms >3 months since injury), and 15 healthy controls (100). Their model was able to diagnose mTBI and PCS participants with an accuracy of 67% and 71%. Their ANN was not able to distinguish PCS patients from acute mTBI, suggesting there were anti-saccade abnormalities persisting in those with PCS (latency and error rate) (100). Unfortunately, however, this ANN has not been used on larger data sets so the generalizability of these results are not known. However, other groups have revealed persistent deficits in PCS patients, most notably in anti-saccades, self-paced saccades, memory-guided saccades, and smooth pursuit function beyond 3 months (74; 77). In Heitger and colleagues’ PCS cohort (within one year of injury, compared to 301 symptomatically recovered mTBIs >6 months post-injury), they noted higher directional errors, reduced performance on memory-guided saccades, reduced self-paced saccades with reduced velocity, and anti-saccade errors (77). Importantly, reflexive saccade measures were not significantly different between groups in any of these measures, implying a cognitive basis for these findings. It must also be noted that these patients were recruited through seeking medical attention for prolonged symptoms and were covered by no-fault insurance for their visits. The authors performed a battery of neuropsychological tests in an attempt to control for any secondary motives.

Logitudinal studies are scarce in eye movement and mTBI literature which make it difficult to ascertain when ocular motor abnormalities begin to resolve and whether this is correlated to a decreased symptom burden in clinical practice. In addition to the groups mentioned above (16; 87; 142), one 12-month study followed a cohort of 37 mTBI patients (more severe spectrum of mTBI with post-traumatic amnesia) over 12 months (6 patients lost to attrition) with measurements at 1 week, 3 months, and 6 months. Acutely, there were increased latencies in anti-saccade and memory-guided saccades with no differences in reflexive saccades. By 6 months, saccade latency and directional errors (during anti-saccades and memory-guided saccades) returned to control levels (after remaining impaired at the 3-month time point). Mean absolute position errors (accuracy) remained impaired at 6 months and recovered by 12 months. Only 16% of patients were symptom-free at 3 months with 14% symptom-free at 6 months and 39% symptom-free at 12 months (75). Heitger’s group published another 37-patient cohort the following year which showed a strong association between impaired ocular motor function and delayed recovery. This was found to be more sensitive than neuropsychological assessments (76).

In summary, saccadic impairment in mTBI shows dysfunction during more cognitively demanding tasks. Reflexive saccades with basic saccade measures (gain and velocity) were poorly sensitive. Most frequently reported impairment in complex tasks were latency, followed by gain and positional errors with scarce pathophysiological evidence from advanced neuroimaging. In addition, eye tracker sampling rates varied between studies, from as low as 60 Hz which is known to affect outcome measures (102), with poorly reported details on precision, accuracy, and calibration. To consider any of these measures as a future biomarker, there is a need for standardization of saccade protocols with global consensus in the eye tracking community. Importantly, Nij Bijvank and colleagues from the Amsterdam University Medical Center and Moorfields Eye Hospital (London) (134) published a standardized protocol for saccade metholodogy and analysis on healthy participants which included and expanded on recommendations from an international expert meeting in 2013 (9). They reported excellent reproducibilty of basic measures (134). However, in more complex tasks (anti-saccades and double-step saccades), their reported reproducibility was low in healthy controls, which questions whether these methods are appropriate for diagnostic use on an individual level. This variability will increase in disease states such as mTBI. In addition, their protocol is 21 minutes long which may limit its adoption, particularly in mTBI patients with symptom provocation and attention deficits (9). Overall, more transparent, robust, and larger studies are required to further investigate this area before saccades are considered ocular biomarkers.

## Smooth pursuit

Smooth pursuit eye movements track moving objects, integrating sensorimotor feedback from multiple brain regions to maintain an image on the fovea (17). Its susceptibilty to mTBI-related pathophysiological change is due to its reliance on the communication (white matter tracts) between widespread gray matter regions. Visual information is relayed to the middle temporal visual area which projects to the medial superior temporal visual area (MST) and frontal eye field (FEF), responsible for reacting to motion. The frontal pursuit area, lying in the posterior FEF, will respond to the specific vectors of motion, providing a signal to facilitate smooth pursuit (105; 106; 171; 172). From the brainstem (pons) and cerebellum, neurons are synchronized with velocity and direction, adjusting themselves accordingly to the stimulus (2; 18; 136; 147; 173). During smooth pursuit, saccades (referred to as ‘catch-up’, and ‘back-up’ saccades) compensate for the velocity of the moving object when fixation is lost (18).

### Clinical Measurement

**Figure 2.**
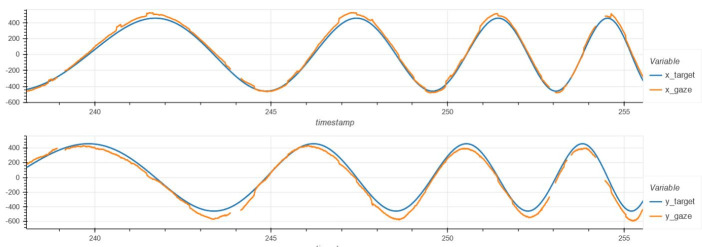
Smooth pursuit measurement on a portable eye tracking device operating at 200 Hz. This healthy participant followed a circular pattern, appearing sinusoidal when X- and Y-axis coordinates are analyzed individually. The blue line represents the path of the target while the orange line represents the participant’s gaze trajectory. Gaps in data represent blinks. X-axis represents time in seconds while the Y-axis represents distance in pixels. Original data acquired using a 200 Hz eye tracking device on a healthy 42-year-old male volunteer.

Smooth pursuit requires the patient to follow a target ‘smoothly’ across their field of vision. They are instructed to follow an object (or fingertip) from one meter as the the target moves half a meter to the right and left at a speed of 2-3 seconds in each direction. This test is repeated again for vertical movements. An abnormal result may be interpreted by the examiner as excessive saccadic interruptions. Smooth pursuits in every direction will also test cranial nerves III, IV, and VI (Figure 2). During the VOMS screening tool, only symptom provocation is recorded (section below) which is not a measurement smooth pursuit quality.

### Smooth Pursuit and mTBI

Multiple groups have studied smooth pursuit eye movements in mTBI patients in both clinical and laboratory settings (10; 35; 46; 49; 50; 55; 77; 92; 109; 114; 148; 163). These patients generally experience a slower tracking velocity compared to healthy controls with a higher mean positional error which has also been found in persistently symptomatic (‘postconcussion syndrome’, PCS) patients (10; 35; 46; 50; 77; 114).

Michael Kelly developed a portable device displaying a 10-second smooth pursuit figure-eight protocol which has been used in 849 athletes (aged 12-18) and 98 mTBIs (92). 42 of these mTBIs had baseline scores. mTBI patients showed grossly skewed pursuit movements based on z-scores from normative data which may show promise for the application of a sideline detection tool on sports fields. A particular strength is their use of post-test coordinate transformation, eliminating the need for a pre-test calibration which reduces acquisition time for participants and potentially increases accuracy.

Maruta and collagues studied a smaller cohort of 13 acute mTBIs within 2 weeks of injury and compated this to 127 normal subjects, in addition to 43 volunteers who underwent sleep deprviation (111). Smooth pursuit followed a circular pattern and was recorded via eye tracking technology. The mTBI subjects showed increase positional error variability, reduced radius of pursuit, and reduced velocities relative to control. Stability of tracking was recorded as standard deviation of radial error (SDRE), standard deviation of tangential error (SDTE), and horizontal/ verical gain (‘H-gain’ and ‘V-gain’, respectively). Sleep-deprived subjects showed impaired SDRE, SDTE, and H-gain. However, mTBI was still significantly worse in SDTE and V-gain. This variability was attributed to impaired predictive timing in mTBI, whereas sleep deprivation revealed variability in radial error from gaze stability degradation only, supported by a previous small primate study (167). With an increasing cognitive load (performing a word-recall task during eye tracking), Contreras and colleagues revealed that mTBI showed significantly altered performance in circular smooth pursuit relative to control, whereas controls showed improvement with the increased cognitive load (considered to increase their attention toward the task) (42). This study differed by measuring synchronization indices and analysed the first and second half of the experiment independently to control for fatigue-related effects. However, these conclusions are limited by small cohort size and lack of symptom evaluation (n=12 mTBI, 2.2±1.8 years post-injury, and 12 age-matched controls).

Wetzel’s group also tested a circular smooth pursuit task in their cohort of 71 military personnel (75 age-similar controls) with post-concussive syndrome, revealing increased intersaccadic intervals and a trend toward increased amplitudes (186). Linear horizontal ramp testing (smooth pursuit at varied speeds) revealed lower gains, and increased fixation durations. For vertical ramp testing, only saccadic amplitude was increased between brain injured compared to controls. Although the investigators do not highlight this difference in their discussion, this may have been attributed to shorter distances traveled in the vertical plane due to a rectangular monitor for stimulus presentation.

Kelly’s high-school group of 50 mTBIs and 170 controls (mentioned in ‘Saccades’ section above) revealed increased latency in initiation of circular smooth pursuit across all timepoints with reduced position gain and velocity gain. However, the significance of this is not clear with no correlation made to symptom severity and time from injury (timepoints post-injury ranged from 1-328 days) (91). Suh and colleagues performed a similar task on 26 mTBI patients: 20 patients within 6 weeks-2 years post-injury, and 6 patients within 14 days of injury (26 controls) (165). Their smooth pursuit task differed through incorporating ‘target blanking’ where the target disappeared to remove retinal input and directly measure cortical input with predictive tracking. Despite the variability in their mTBI cohort, these patients revealed shorter times to saccade iniation on target blanking, increased intra-individual variablity, greater ocular motor tracking error before and during target blanking, and greater lag.

DiCesare and colleagues studied an acute cohort of 17 acute mTBI patients within 7.6 days (+/- 4.7) of injury. These patients showed higher phase lag times (slower response to stimulus, most dramatic at higher speeds) and reduced velocities (49). This was also supported by Evans and colleagues who found increased mean error during faster smooth pursuit conditions (n=26, average 32 days post injury +/- 37) compared to healthy counterparts (55).

Johnson and collagues studied a small cohort of 9 ‘recently concussed’ (time since injury not reported) patients who performed both circular and sinusoidal smooth pursuit, in addition to fMRI (88). Although this group noted abnormal horizontal saccades, self-paced saccades, anti-saccades, and memory-guided saccades, they did not find a significant group-difference in smooth pursuit. However, these tasks were associated with widespread activation of brain areas relative to controls which was considered to be due to the compensatory recruitment of other brain regions to assist with tasks (reactive collateralization and ‘increased brain effort’). Specifically, these were the cerebellum, bilateral secondary visual cortices, and the V5/MT visual area. At 30-day follow-up, saccade metrics improved (albeit still impaired relative to control) and smooth pursuit remained insignificant (87). However, on fMRI, extrastriate visual area V5/MT remained hyperactivated with activation decreasing (from initial scan) in the cerebellum, precuneus, superior temporal gyrus, hippocampus, and postcenral gyrus. The significance of this requires further reseacrch and the results of this should be interpreted with caution due to their underpowered sample size and lack of test-retest measures.

Richard and colleagues tested a cohort of recently concussed (sports-related) and historically concussed (1-5 years post-injury) athletes. They found the acutely concussed experienced worse impairment in vertical and circular smooth pursuit which remained impaired in the older mTBI group, suggesting the smooth pursuit impairments persist beyond symptomatic recovery (149). Likewise, Cochrane and colleagues’ cohort (mentioned in Saccades revealed similar results: decreased vertical position gain relative to controls which was only significant at increasing speeds (38).

### Smooth Pursuit in ‘Postconcussion Syndrome’

Smooth pursuit has also been extensively studied in postconcussion syndrome (patients with an ongoing symptom burden). Diwakar and colleagues studied smooth pursuits in a cohort of 25 ‘chronic mTBI’ (3 months to 5.5 years post-injury) (50). mTBI patients revealed a larger radius around the target of pursuit (less accuracy), and when the target disappeared and reappeared along the radius of the circle, they were less likely than controls to predict its location and resynchronize with the target, suggesting a deficit in anticipatory control. Using magnetoencephalography (MEG), they revealed impaired beta activity (alpha, beta, and gamma-band oscillations were tested). This was suppressed in the parietal cortex (particularly right parietal) and increased in the left caudate and frontal-temporal regions. This was shown to be 92% sensitive for mTBI diagnosis using their model. However, test-retest reliability was not reported and these results have not been replicated, questioning its feasibility and reproducibility.

Heitger and colleagues performed another study in PCS patients (n=36, within one year of injury) to healthy counterparts. The impairment of smooth pursuit was positively correlated with symptom severity. These patients generally showed significantly higher mean error in pursuit, with lower peak velocities and higher lag (77). A further cohort of 36 mTBI patients averaging 43 months post-injury (+/- 52 months) revealed a higher number of saccadic intrusions with reduced accuracies and slower reaction times during a sinusoidal smooth pursuit paradigm (46).

Astafiev and colleagues used a case-control study design across two centres (45 chronic mTBI patients >3 months post-injury) which aimed to correlate smooth pursuit to functional MRI signals (blood-oxygen-level-dependent; BOLD) and diffusion MRI (DTI) (10). There were no between-group differences in smooth pursuit tracking error which was considered to be due to heterogeneity in injury severity between the two centres, and different eye tracking apparatuses. However, they noted significant differences in white matter regions, notably the anterior internal capsule and superior longitudinal fasciculus. This was consistent with abnormal BOLD signal in these regions, albeit varied across individuals.

In terms of smooth pursuit error and correlation to symptom burden, Cifu et al. studied 60 chronic mTBI patients (military cohort with confirmed loss of concioussness) and found larger mean error in target pursuit which was associated with greater symptom severity (35). Maruta and colleagues revealed similar findings (114). 17 chronic PCS patients (>6 weeks post-injury) showed a higher gaze error which positively correlated to severity of attention and working memory. This was also correlated to reduced integrity of the right anterior corona radiata, left superior cerebellar peduncle, bilateral uncinate fasciculus, forceps major, and the genu of the corpus callosum on diffusion MRI (DTI). In two further studies, this group studied a 32-patient mTBI cohort (3 months to 5 years post-injury, milder to previous cohort) with the same circular smooth pursuit trajectory, and correlated once more to DTI. This second study did not find any significant difference in white matter tracts or smooth pursuit eye movements which was considered to be due to milder injury and less symptom burden, meaning patients may have experienced a greater degree of recovery in this study (112). They published a second part of this study which performed the eye tracking once more after an attention-demanding task. With this protocol, there was increased variability in smooth pursuit performance, suggesting an increased level of task-related fatiguability in mTBI patients (113).

Stubbs and colleagues also assessed smooth pursuit with an increased cognitive load. Their cohort of 16 persistently symptomatic mixed-cause mTBI patients (within two years post-injury, mean 5 months, compared to 15 age- and gender-matched controls) undertook a baseline smooth pursuit task followed by the same task during a working memory task (*n-back)* (163). In addition to slowed reaction times and increased errors in the *n-back* task, the mTBI group did not improve their radial (and overall) pursuit variabilitiy with increased working memory load when compared to controls. Baseline smooth pursuit without a working memory task showed a group-wise diagnostic performance of 58% (radial variability) which was increased to 79% with the concurrent working memory task. Overall, this study provided further evidence of reduced attention following mTBI which, when combined with an eye tracking task, increased diagnostic sensitivitiy. It is worth noting, however, this study’s small sample size, in addition to 31% of participants being eligible for compensation claims, may have lead to a potential performance bias.

In summary, a majority of studies suggest impaired smooth pursuit in mTBI, most commonly described as increased lag and decreased accuracy which is correlated to higher symptom burden and injury severity. In studies where no differences in smooth pursuit metrics were found, it was suggested that mTBI was on the milder spectrum with limited studies showing abnormal neuroimaging findings and larger inter-individual variability in smooth pursuit measures. Studies did not report participants’ calibration which is a key variable in calculating smooth pursuit accuracy with gaze coordinates. Additionally, lack of standardization of eye tracking protocols and patient cohort demographics (including time post-injury) make it difficult to compare results across studies. The effect of cognition is an important factor in smooth pursuit which influences its potential as an ocular biomarker. More research is required to understand which aspects of cognitition (e.g. working memory load or attention) are most effective in increasing the sensitivity of smooth pursuit. Following this, larger trials evaluating the diagnostic efficacy of such protocols must be performed.

## Vestibulo-ocular reflex (VOR)

The vestibulo-ocular reflex stablizes an image on the fovea during rapid head movement. VOR is composed of three components: peripheral inputs from vestibular organs (semicircular canals and otolith organs of the inner ear), central integration through widespread cerebral pathways (cerebellum, vestibular nuclei, oculomotor nuclei, thalamus, spinal cord autonomic system, and contralateral nuclei), and motor outputs (e.g. extraocular muscles) (45). The VOR response is regulated by the cerebellum (inferior cerebellar peduncle, flocculonodular llbe, and fastigial nuclei) and includes widespread projections through the greater cerebral cortex, thalamus, and reticular formation to allow for spatial awareness (160). mTBI may broadly affect the vestibular system in one of two ways: peripherally and/or centrally. For example, pressure waves from blast-induced mTBI cause trauma to the inner ear which damages peripheral vestibular sensory organs (semicircular canals and otolith organs) (93). Likewise, mTBI-induced pathophysiological change to the cerebellum (e.g. diffuse axonal injuries and microhaemorrhages) leads to central vestibular dysfunction (6; 63).

### Clinical Measurement

A clinician should be aware that the vestibulo-ocular reflex (VOR) will often provoke symptoms when tested on mTBI patients (129). First, the examiner must ensure there is no cervical spinous/ muscular injury. For horizontal VOR testing, the patient must rotate their head horizontally by 20 degrees as quickly as they can while focusing on a target placed approximately 1 meter away from them (5). This may also be performed by the examiner placing both hands on the patient’s head to perform this test manually. A more precise method consists of a rotational chair, but even these are not sensitive enough for unilateral vestibular hypofunction (only bilateral) (45). A failed VOR test results in failure of the eyes to remain on target. This may be as subtle as a small corrective saccade after the movement. Vertical VOR is tested by asking the patient to move their head vertically (moving their head up and down by 20 degrees whilst maintaining focus on a target). The video head impulse test (vHIT) is a more objective method which uses a commercial video-oculography (eye tracking) headset and records saccades and horizontal VOR gain (128). A separate test, referred to as the caloric test, stimulates vestibular sensory cells to activate efferent ocular motor nerves via the VOR. This consists of instilling cold or warm water in the external auditory canal. Cold water will cause a head turn and horizontal nystagmus to the contralateral side (with eyes turning to the ipsilateral ear) due to decreased vestibular afferent firing. Warm water will cause a head turn and horizontal nystagmus to the ipsilateral side (with eyes turning to the contraleral ear) with increased firing rate of the vestibular afferent nerve (56). However, this test is limited to stimulating only the horizontal semicircular canals at low frequencies (daily head movement is high frequency along multiple angular planes) which is a significant limitation (20; 70; 141). Even in patients with peripheral vestibular dysfunction and known canal dysfunction (paresis), the sensitivity is poor with a high false positive rate (20). In the setting of an abnormal caloric test result, one group argues that VOR is also likely to be abnormal which may give positive predictive value, but still warrants further vestibular testing (182). In chronic vestibular complaints, this is less clear with evidence to suggest the opposite (VOR was frequently reported as normal in patients with abnormal caloric testing) (126). Nevertheless, this is rarely performed in a clinical setting with mTBI patients (i.e. concussion clinics) and these measures do not conclusively test every aspect of the vestibular system, nor connect all components of the ocular motor system which limits their utility (39). Instead, a more common tool is used by mTBI practitioners: the Vestibular/Ocular Motor Screening (VOMS) tool which rates symptom provocation following a series of eye movement tasks (see Table 1). This test was developed as a brief screen to assess vestibular and ocular motor impairment in mTBI patients (129).

**Table 1:** Vestibular/Ocular Motor Screening (VOMS) tool, adapted from (5)

Baseline Symptoms	Headache 0-10	Dizziness 0-10	Nausea 0-10	Fogginess 0-10	Comments
Smooth Pursuits					
Saccades – Horizontal					
Saccades - Vertical					
Convergence (Near Point)					(Near Point in cm):
					Measure 1:
					Measure 2:
					Measure 3:
VOR – Horizontal					
VOR – Vertical					
Visual Motion Sensitivity Test					

The VOMS tool was originally reported to have a sensitivity of 50% for item symptom scores ≥ 2 (129) with a recent study of adolescent sport-related concussion suggesting it may be useful for identifying those with prolonged recovery (>30 days), albeit with a high number of false positives (95).

### VOR and mTBI

In nearly all studies evaluating VOR in mTBI, the majority comment on symptom provocation during testing (e.g. symptoms of nausea following rotational acceleration during VOR testing) which does not provide any empirical evidence of VOR dysfunction (symptoms may be caused by non-vestibular disorders). However, in a series of early studies, patients complaining of vestibular symptoms (dizziness and vertigo) from mild to moderate TBI have been shown to have abnormal caloric testing in 3-40% of cases (22; 62; 180). A more recent study of 27 blast-induced mild to moderate TBI veterans showed positive vestibular findings in 50% of those experiencing dizziness as a symptom post-injury.

However, higher reported symptoms on testing may correlate to a longer recovery time which is clinically relevant (54; 115). Babicz and colleagues examined 158 participants (age 16.5 +/-2.8) which correlated high VOMS scores to symptom severity. Women reported higher symptom scores than men on VOR testing. Positive symptom provocation on vertical VOR testing was independently associated with a high post-concussive symptom score (details of whether or not the reflex was abnormal was not reported, nor were prevalence of vestibular symptoms) (13). Mucha and colleagues evaluted 64 mTBI patients (age 13.9 +/-2.5) within 5.5 (+/-4 days) of sport-related mTBI (compared to 78 controls) (129). They assessed horizontal VOR clinically and found 61% experienced symptom provocation (abnormal VOR response not specified, nor were specific symptoms). Their follow-up study examined 36 male and 28 female athletes aged 9-18 years within 21 days of sport-related mTBI (164). Females not only experienced higher post-mTBI symptom score, but also scored significantly higher on symptom scores following VOMS and VOR which is considered to be in part due to differences in neck muscle bulk. Schneider and colleagues evaluted 559 elite-level ice hockey players (aged 13-17) pre-season and post-mTBI (155). Before the start of the season, 3 players showed a unilateral positive head thrust (VOR) test (potential false positives). During the season, 97 players (17%) suffered an mTBI, of which 8 (8% of those injured) showed clinical VOR dysfunction. 23 of these players (24% of those injured) experienced dizziness as a symptom. Although this is in line with previous estimates of vestibular dysfunction in those reporting dizziness as a symptom, readers would have benefited from further evaluation of vestibular dysfunction.

Five notable groups have quanititatively measured VOR using the video head impulse test (vHIT) headset which quantifes corrective saccades (indicative of an abnormal response) following the rotational movement performed by the examiner. Alkathiry and colleagues examined 25 symptomatic adolescents (ages 12-19) within 10 days of sport-related mTBI. Measures of VOR gain (i.e. vHIT gain) in all patients were considered to be within normal limitis, even while using the quantitative video head impulse test, despite high scores on the VOMS symptom report (7). This supports the notion that symptom provocation is poorly sensitive to vestibular dysfunction. Another group, Alsheri et al., examined 56 mTBI subjects (29 aged <21, and 27 adults age 21-68) of a mean 4 months post-injury for <21 and <6 months for adults (8). Their group did not reveal any abnormal vHIT findings, but headache, dizziness, and nausea were significantly worse post-vHIT testing. Ellis and colleagues compared 48 adolescents (aged 13-18) with an mTBI in the past year to 165 athletes without a history of mTBI in the past 12 months. They also did not find any between-group differences in VOR function using a vHIT headset (54). However, Balaban’s group used a computer-controlled, rotational head impulse test in 100 acute mTBIs which showed significantly decreased gain and asymmetry between each eye’s response compared to their 200 controls (16). This persisted at 2 weeks, particularly in those symptomatic (78).

In the past decade, ocular vestibular evoked myogenic potentials (oVEMPs) have emerged as a means to evaluate utricular otolith function (cervical VEMPS mainly evaluate saccular otolith function) (41; 108; 183; 184). Using air-conducted sound or bone-conducted skull-based vibration, signals from the inferior oblique are recorded via electrodes placed below each contralateral eye. During VOR provocation, signals from vestibulo-ocular projections from the otoliths are quantified, providing a more precise measure of function, especially during vHIT (108; 183). Two studies have evaluated oVEMPs in mTBI cohorts (123; 149; 187). Rodriguez and colleagues studied an asymptomatic paediatric cohort (51 mTBI 3 months to 7 years post-injury and 25 controls, mean age 16) of which 20 previous-mTBI patients were in non-contact sports and 31 in contact sports. Sustained upgaze along with head thrust testing was performed. Abnormal or absent oVEMP responses were more prevalent in contact-sports athletes than non-contact athletes with a history of mTBI, particularly when compared to controls. This was considered to arise from the susceptibilty of the utricle’s relatively weak support in the temporal bone, making it more vulnerable to repetitive trauma (149). Meehan and colleagues also showed prolonged oVEMP latencies, but in a cohort of 71 military service members with previous mTBI (75 normative controls). Otolith dysfunction was more pronounced in mTBI participants with anxiety, depression, and post-traumatic stress. The authors hypothesized this to be due to a multisensory mismatch and highlighted shared neural pathways between the vestibular system and emotion processing (110). The significance of this and causal relationship requires further study. Additionally, the extent of oVEMP dysfunction in other mTBI subtypes is not known.

Overall, the majority of VOR studies were only able to describe symptom provocation with all but one reporting abnormal VOR responses. These studies are limited by a failure to evaluate other domains of vestibular function to validate their claim of ‘abnormal’ or ‘normal’ VOR given its high false positive rate. Symptoms (and their provication) are a poor indicator of vestibular dysfunction. Emerging evidence of more precise measures of otolith function, such as oVEMPs, show promise for future ocular biomarkers, but this area is in its infancy and requires further research. To reach the stage of being classified as an ocular biomarker, abnormal VOR responses must be measured more precisely with higher reproducibility, such as through the combination of vHIT testing and oVEMPs. To reach this stage, standardized procedural and analytical methodologies are required. This requires larger longitudinal studies to evaluate its generalizability, sensitivity, and specificity which would precede studies evaluating its diagnostic utility.

## Vergence

Vergence ocular motor functions occur from non-conjugate movements in eye position to view subjects near or far (166). Binocular fusion on a near target occurs through bilateral adduction of the eyes (convergence) while abduction of the eyes provides clear distance vision (divergence). In mTBI, a purely speculative theory of vergence impairment arises from a ‘global processing delay’ of afferent pathways, suggested by increased latency and decreased velocity for both convergence and divergence (175). This results in less signal input to dedicated brain regions, identified in non-human primates as the midbrain supraoculomotor area (47), frontal eye field (161), supplementary eye field (157), superior colliculus, pretectum, accessory optic nuclei (27), and cerebellum (61).

### Clinical Measurement

There is currently no means of precisely measuring a vergence index between the two eyes from moment to moment as a patient performs a task. In a clinical setting, divergence is measured by placing an object close to a patient’s face until they lose fusion and see two objects (diplopia) using a horitzontal base-in prism bar (negative fusional vergence range) (146). Convergence, by contrast, is tested by asking a patient to focus on an accommodative target, best measured using Beren’s ruler. The examiner moves the target toward the patient until a deviation in one eye occurs (1). A precise step-by-step procedure for near point convergence testing has been summarized by the Convergence Insufficiency Treatment Study Procedures Manual by Scheiman and colleagues (69).

Convergence insufficiency (CI) is a term used to describe when this fails which fulfils criteria and must meet the criteria in Table 2.

**Table 2:** Criteria for convergence insufficiency adapted from Raghuram and colleagues (146)

Mandatory criterion:	In addition to at least one of the below criteria:
Exophoria at near (4 prism diopters or larger in magnitude compared to distance)	Receded (abnormal) near-point convergence (generally >7cm)
	Reduced positive fusional vergence* (convergence amplitudes of greater than or equal to 15 prism diopters of break/ Sheard criterion not met). However, this may require adjustment based on age (152)
	Vergence facility of less than 9 cycles per minute with poor fusion of base out prism

Convergence fusional amplitude is the magnitude of prism a patient can tolerate before they experience diplopia (e.g. introducing a base out prism over one eye whilst the patient focuses on a central target).

Table 3 illustrates normal values which are unaffected by refraction at population-level (137). NPC distance has been shown by one group to be, on average, double (200% of normative value) in mTBI patients (175).

**Table 3:** Near Point convergence values in centimeters by age (137)

Age	Percentile 85%
10-19	10
20-29	11
30-39	12
40-49	15
50-59	15
60-69	20
>70	20

### Vergence and mTBI

Although the prevalence of CI in the non-presbyopic normal population is reported to be 2-13% in varied populations (and diagnostic criteria) (71; 145; 150), its combined prevalence increases to 37.2% in TBI (95% confidence interval, 24.3 to 51.1%) as reported in a recent systematic review and meta-analysis (125).

CI is also correlated to a high symptom burden in those with mTBI. In a cross-sectional study of 34 adolescents (aged 9-17) with a recent diagnosis of mTBI, attention, learning, and memory were significantly worse in those with convergence insufficiency (140). In this group, 80% had visual symptoms (78% with vergence disorders, 48% with accommodative impairment, 41% with true CI, and 41% with ocular motor disorders). Half of their participants had overlap between all of these issues. In a more severely injured cohort of moderate-severe TBI (n=26), convergence insufficiency was positively correlated to coma duration, lasting cognitive disruption, decreased incidence of return to work, and over-all poorer rehabilitation outcomes (40). Tyler and colleagues (181) measured both divergence and convergence in a cohort of 12 mTBI patients (range 2.4 months to 35 years post-injury; mean 2.2 years with 9/12 persistently symptomatic). In addition to slowed velocities of vergence (convergence relative to divergence) they revealed reduced activation of the lateral geniculate nuclei, superior colliculi, oculomotor nuclei, supra-oculomotor areas (found to be most sensitive), and abducens nuclei (181). A retrospective neuroimaging study of 25 mTBI patients (median time from injury: 20 days; range 1-486 days) with convergence insufficiency (compared to 17 mTBI patients with normal convergence) showed abnormal diffusion MRI parameters (disrupted fractional anisotropy) to the right anterior thalamic radiation and right geniculate nucleus optic tracts which also correlated to decreased processing speeds (6). However, their analysis was not blinded and baseline differences between groups were not known. Larger cohorts evaluating these regions with strict time points are required to clarify the validity of this result.

Near-point convergence (NPC) distance alone is increased (abnormal) in a large portion of mTBI patients (26; 31; 139). This has been shown to be even more prevalent in patients who are persistently symptomatic, averaging 7 months post-injury until recovery (146). Abnormal NPC is also correlated to worse verbal memory following mTBI with reduced visual motor speed, reduced reaction time, and higher symptom burden (139), making it a useful marker of severity and recovery time (3; 116; 169). In contact sports players sustaining repetitive, sub-concussive impacts (as measured via mouthguard accelerometers), two studies have shown increased NPC distance most marked mid-season, resolving by 3 weeks post season (101; 188). In a paediatric cohort aged 12-17, impaired NPC of over 6cm was positively correlated to persistent mTBI symptoms and correlated to more subtle abnormalities on high frequency eye tracking (24). This study trained a statistical model on a small cohort of 51 controls and 24 mTBI using vergence metrics (measured via binocular eye tracking as the difference between each eye’s position from second-to-second) which showed a sensitivity of 75% and specificity of 64.7% in determining mTBI from non-mTBI. This metric was then able to classify patients based on the NPC status with a specificity of 95.8% and 57.1%. This form of study moves closer to the useful application of this technology in revealing a useful combination of biomarkers.

In a blast-induced mTBI cohort (mean time from injury: 4 years), 25% were found to still have convergence insufficiency (109). Kowal and colleagues revealed in a 164-patient cohort of TBI’s (unspecified severity), that 14% experienced convergence insufficiency. Of these, 35% of patients persisted beyond the 12-month follow up (98). Divergence appears less explored in both clinical practice and in the literature.

In summary, limited evidence suggests impairment of the vergence response in mTBI. Prognosis is mixed with many patients persistently symptomatic in months to years after their injury. For consideration as a future biomarker, it may be useful to combine this measure (and correlate) with other ocular motor assessments as an overall weighted score. However, to reach this level of diagnostic efficacy on an individual level, larger longitudinal trials are required to control for age-related effects, time post-injury, and severity of injury.

## Pupillary Light Reflex

The pupillary light reflex (PLR) is dependent on both the parasympathetic nervous system for pupillary constriction and the sympathetic nervous system for pupillary dilation (21; 103). Its role is to balance visual sensitivity and acuity through its autonomic nervous system integration (120). The afferent pathway sends neuronal impulses from the retinal ganglion cells to the superior colliculus and pretectal area of the midbrain. From here, bilateral impulses are received by the preganglionic parasympathetic nuclei (in the midbrain, known as the Edinger-Westphal nuclei) along with the hypothalamus and olivary pretectal nucleus. Efferent fibers innervate the oculomotor nerve and ciliary ganglion (preganglionic), directly innervating the iris sphincter muscles, causing pupillary constriction (52; 90; 185). Its sympathetic pathway travels from the hypothalamus to the brainstem and spinal cord to the superior cervical ganglion at the bifurcation of the carotid artery, sending postganglionic fibers to the dilator pupillae muscles via ciliary nerves (151). In mTBI, it is not known whether autonomic dysfunction or structural damage to these areas are related to differences observed between healthy participants and mTBI patients.

### Clinical Measurement

In a dim light, a pen torch is shone at a straight angle directly into the patient’s eye, ensuring no light contamination to the fellow eye. The light is then withdrawn for a few seconds, followed by a repeat attempt, but observing the response of the fellow eye (indirect, consensual pupillary light response). This is measured on a 0 to 4+ grading scale, where a healthy individual has a brisk, responsive 4+ score, with 3+ indicating a moderate response, 2+ slowed, 1+ barely visible contraction, and 0 unresponsive. Pupils are approximated in millimeters using a ruler (21).

### Pupillary Light Reflex and mTBI

In mTBI, potential decreases in neurosensory gain from resulting injury may provide less signal through the afferent system to drive pupillary constriction, but this has yet to be proven. This reflex has been shown in select studies to be altered in mTBI which is most readily appreciated through objective evaluation using eye tracking technology and pupillometry. Although evidence is scarce, select investigators have shown that dynamic velocity impairments are detectable in mTBI.

Capó-Aponte and colleagues used a monocular infrared pupillometer in a large case-control study of 100 military personnel with acute mTBI (<72 hours) and 100-age match controls (29). They found that acute mTBI patients had slower average pupillary constriction and dilation velocities and slower 75% recovery times (the total time for the pupil to recover 75% of its initial resting diameter following constriction). The same investigators also observed that constriction latency, average constriction velocity, dilation velocity, and 75% recovery time were all significantly impaired in the mTBI group between 15-45 days post-injury.

Similarly, sports-related concussion patients have abnormal pupillary responses. A larger cohort of 135 athletes (aged 14-18) were followed throughout a sports season where 7 mTBIs were reported. By measuring the response to the pupillary light reflex over 5 seconds (0.8 seconds of bright white light, 150 lux), they showed an ‘enhanced’ (brisk) light reflex on the day of the injury with a marked reduction (constriction and dilation velocities) during the recovery process (days to weeks) (144). A longitudinal cohort study examined 18 high school football athletes, pre-, mid-, and post-season, in addition to when athletes experienced a high-acceleration head impact (as measured via helmet impact accelerometry during matches). Athletes with both concussive and ‘sub-concussive’ impacts (termed ‘asymptomatic high-acceleration head impacts’, categorized by >95*g* of linear acceleration and >3760 rad/sec^2^ of rotational acceleration) showed deceased pupil dilation velocity, alterations in resting pupil diameter and decreased constriction velocity. This was not associated with a significant change in symptom scores. Over the course of the season, constriction velocity was also significantly decreased, which suggests that pupillary function may serve as a sensitive tool for sub-concussive impacts and ‘sub-clinical’ brain trauma (85).

mTBI patients with an ongoing symptom burden (‘chronic mTBI’ or PCS) may also have abnormal pupillary responses. In a small cohort of 17 chronic mTBI patients (the majority from road-traffic accidents) at one year post-injury, participants were found to have reduced constriction velocity, dilation velocity, and amplitude of constriction. The authors suggested slowed dilation metrics with reduced maximum pupillary diameters were from sympathetic dysfunction, whereas the reduced peak velocities (and amplitudes) were due to parasympathetic involvement to a lesser degree (174). This study is problematic for a number of reasons. There was a selection bias toward persistently symptomatic patients (medicolegal status not reported), in addition to lack of age-matching (the authors report a 17% group difference being explained by older age in the mTBI group. In addition, one participant had a history of migraine which has been shown to affect pupillary dynamics (44).

Truong and colleagues, at the same institution with a co-author from the previous study, also examined a cohort of chronic mTBI patients (32 subjects >45 days post-injury of mixed-cause mTBI) who were referred with visual complaints (medicolegal status not reported) and compared them to 40 controls (age-similar only) using pupillometry-measured dynamics with a red, white, and blue light stimulus (178). Across nearly all conditions, mTBI patients showed increased constriction latency, reduced pupillary diameter, reduced constriction velocity, reduced amplitude of constriction, and reduction in 6 seconds post-stimulus diameter to less intense light stimuli. Across both groups, blue light showed a marked delay in dilation (a sustained constriction response due to the melanopsin-expressing intrinsic photosensitive retinal ganglion cells-driven pupil response (138)), but the effect was most pronounced in the mTBI group. However, these results may require interpretation with caution: participant details were scarce with no ocular biometry or days post-injury reported.

Truong et al (177) later investigated the key role refraction and biometry plays in pupillometry, with high myopes experiencing the slowest velocities, presumably due to altered biomechanics (e.g. tissue elasticity) and reduced sympathetic drive (a key driver for refractive state) (64) which has implications as a potential confounder in these studies. The same investigators validated the utility of monocular pupillometry by demonstrating that pupillary responses in normal and mTBI patients are symmetrical (179).

In summary, there is scarce evidence to suggest pupillary constriction latency, constriction/ dilation velocity, and recovery time are delayed in mTBI patients. More information is required on how individual and experimental factors (e.g. age, biometry, refraction, task, and background luminance) influence pupillary responses. In addition, important confounders exist in the pupillary response such as perceptual awareness (53; 133), attention (25; 117; 118; 132), mental imagery (99), eye movement preparation (119), pain (4; 23), anxiety (23), arousal (11), opioid analgesics (96), alcohol (89), anxiety (89), history of migraine (44), and cognition (19; 143), as a non-exhaustive list (120). For further investigation as an ocular biomarker for mTBI, experimental conditions require meticulous control of these factors prior to substantiating any diagnostic utility.

## Accommodation

The optical power of the eye is controlled via the process of accommodation which brings an image into focus at the fovea through lens thickening (via ciliary body) and pupillary constriction (97). The ciliary zonules contract via the actions of the ciliary body for near distance (lens thickening) and relax for far objects (lens flattening). Three areas compose this circuit: the afferent limb (optic nerve through lateral geniculate nucleus to the occiptal lobe), efferent limb (short ciliary nerves from the Edinger-Westphal nucleus and oculomotor neurons for convergence), and oculomotor control neurons between the two limbs, primarily responsible for transferring the diopteric error (i.e. blur) into the motor command (influenced via visual association cortex and supraoculomotor nuclei) (122) as mentioned in (60). There are currently no studies which examine the aetiology of accomodative dysfunction in mTBI with the exception of case reports on extreme pseudomyopia which are mentioned below.

### Clinical Measurement

Accommodative function is measured clinically by amplitude of accommodation (AA). A common formula estimates one’s accommodative ability in dioptres:

**(1):**
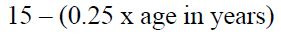


This is tested in clinic using an accommodative rule where a line (or high contrast 20/30 letter) is slowly advanced toward one eye (other eye covered). The point at which the target becomes blurred in front of the eye is read in centimeters which are converted to dioptres (43; 51). Accommodative insufficiency must fulfill one of the following criteria: amplitude of accommodation ≥ 2 diopters below mean for age; monocular accommodative facility ≤ 6 cycles per minute (cpm) (difficulty with minus lenses) (59). Cycles per minute (cpm) refers to the number of times a stimulus is able to be fused (or focused on) through alternative base-in and base-out prisms (58).

Accommodative excess (‘spasm’ with over-contracture of the ciliary body; termed ‘pseudomyopia’) is defined as monocular accommodative facility ≤ 6 cpm (difficulty with plus lenses), whereas accommodative infacility is defined as monocular accommodative facility ≤6 cpm (difficulty with plus and minus lenses) (59).

### Accommodation and mTBI

Accommodative insufficiency may occur post-mTBI, followed by either accommodative excess (pseudomyopia), or dynamic accommodative infacility (i.e. slowed and irregular accommodation) (116). However, dynamic accommodative infacility is poorly described in the literature as this is not readily quantifiable without the use of specialized equipment and expertise.

In non-presbyopic mTBI patients (i.e. preserved accommodative ability), a significant portion of patients may experience accommodative insufficiency, particularly in paediatric cohorts (24; 116). However, true estimates of prevalence are not available due to the majority of studies over-estimating based on small cohorts, referral bias, and mixed severity of injury (65; 121). A meta-analysis of mixed-cause and severity of TBI cites 43% which may still be an overestimation for the same reasons (125).

In a review of 51 pre-presbyopic TBI patients (severity and location of impact not specified) with vision based symptoms (higher probability of selection bias), 41% were shown to have accommodative dysfunction. The majority of these were accommodative insufficiency, but 2 patients had accommodative infacility while 2 experienced accommodative excess (36). The same group performed a laboratory analysis of 12 pre-presbyopic mTBI patients with visual symptoms (time post-injury not known) using an infrared open-field autorefractor which took 5 samples per second over 120 seconds (66). Compared to a control group, all TBI patients showed increased time to accommodate and decreased peak velocity. Responses were slowed up to 4 times normal with significantly varied amplitudes. A third test of accommodative fatigue was employed where the participants were forced to accommodate alternating +1.00 and -1.00 lenses every 10 seconds over 3 minutes. This “flipper rate” was slowed in the TBI group (i.e. these patient took longer to accommodate to each lens as they were presented). In addition, all but two of these patients experienced significant fatigue following the 3-minute session (66). However, this study must be interpreted with caution due to pharmacological confounds in the patient cohort, selection bias, mixed-cause TBI and inclusion of other-cause acquired brain injury (e.g. overdose, encephalopathy), and participation in vision therapy (further bias in interpretation of results).

In general, the prognosis of accommodative dysfunction is largely unknown due to a paucity of follow up data in the literature. A Swedish study of 15 mTBI patients found half to have persistently impaired accommodative ability at follow up (81-322 days), unlike convergence dysfunction which recovered (121). A cross-sectional blast-induced mTBI cohort revealed accommodative insufficiency in 23% of service men at an average of 4 years post-injury (109). In a study of 500 American military personnel, 33.6% of blast-induced and 37.7% of non-blast-induced mTBI patients showed accommodative issues (insufficiency, infacility, or block) which persisted beyond one year (7.6% of blast-induced mTBI patients and only 3.7% of non-blast-induced mTBI patients recovered accommodative facility) (30). A large retrospective analysis of vision therapy in 218 mTBI patients showed that 42% (92 patients) experienced accommodative insufficiency. 39 of these patients showed improvement in their accommodative amplitude over an 18-month period. The other 53 patients were lost to follow up or were not included in the analysis, potentially because they were not satisfied with treatment (i.e. did not see improvement) or went elsewhere (59). Therefore a true indication of accommodative recovery post-mTBI remains under explored.

Pseudomyopia, known as a myopic shift following traumatic brain injury or blunt force trauma to the eye, is largely anecdotal with few studies exploring its aetiology (81; 83; 94; 104; 156; 162). However, two groups (83; 84; 156) have found evidence of ciliary spasm with ciliochoroidal effusions (diagnosed via ultrasound biomicroscopy) causing forward displacement of the lens and shallowing of the anterior chamber which lessens with pharmacological cycloplegia. These more pronounced cases resolved within two weeks, but other cases have shown to persist (81). Other proposed mechanisms (in the absence of biomicroscopy findings) include damage to the accommodative portion of the parasympathetic third nerve subnucleus or disinihibtion of brain stem centres (34), but this has not been scientifically investigated and remains unsubstantiated.

In 1992, a study performed in a rehabilitation unit (unspecified severity of head injuries) of 164 patients revealed 19% had pseudomyopia, in which 55% persisted. These patients, previously documented as emmetropic, complained of blurred distance vision which was amenable to a minus lens (98). Likewise, their reports of impaired accommodation in 16% of patients showed that 58% persisted beyond follow up.

In summary, despite some evidence for impaired accommodation in mTBI patients, it remains mixed with many studies of poor methodological quality. The majority of groups used clinical measurements (prone to subjectivity) and contained selection bias. Dedicated studies with a focus on accommodation (rather than a reported measure) would elucidate the utlity of this ocular measure which remains limited to pre-presbyopic populations.

## Limitations

This review examined promising ocular biomarkers in saccades, smooth pursuit, vergence, VOR, pupillary light reflexes, and accommodation. Across the majority of studies, there were a series of methodological flaws: demographics were frequently lacking with mTBI sub-type, pre-morbid mental health status, inclusion/ exclusion criteria, and severity on presentation not reported. Larger cohorts would enable subgroup analysis of age deciles, gender, and ethnicities (or cultural backgrounds) which may influence these outcomes. Additionally, larger numbers of participants will facilitate symptom correlation (often poor), inclusion of advanced neuroimaging findings (to understand underlying pathophysiology), and recovery risk factors (i.e. which ocular biomarkers on presentation lead to worse prognosis).

A further significant limitation of the current literature is an understanding of generalizability of the reported findings in mTBI, in part due to heterogeneous definitions and criteria. In addition, the relationship between sports-related mTBIs and other causes of mTBI require further clarification. Another relevant issue in mTBI research is whether the differences identified in ocular motor measures are influenced by other factors such as impaired cognition, attention deficits, anxiety, depression, and post-traumatic stress disorder which are recognized to be more common in those who do not recover from mTBI.

Eye tracking methodology was mixed given lack of standardization in current practice. Precision, accuracy, sampling frequency, and calibration details for each participant were rarely reported which may deliver misleading results. For example, a poorly calibrated participant performing a smooth pursuit task assessing accuracy will show decreased accuracy irrespective of pathology. When sampling frequency was reported, select studies reported outcome measures not reliably detectable with such low frequencies (38; 46; 49; 87; 88). Equally scarce were methods of stimulus presentation (e.g. velocity of smooth pursuit target) and analysis (e.g. thresholds for velocity detection). Blinks were not quantified (this may serve as useful data), nor was justification for omitting trials. When deletion of data was mentioned, arbitrary thresholds (e.g. 20% data loss for trial deletion) were reported. In terms of outcome measures, a majority of groups took a broad approach (i.e. multiple outcome measures) which is prone to multiple comparison bias.

There were few studies in real-world community sport, where the majority of mTBIs are not reported and are therefore under investigated (15; 124; 168). Many of these studies took place in dedicated concussion clinics where patients were referred after presenting to health services. This selection bias may increase the prevalence of reported visual symptoms. There is also a paucity of literature on the effect of sub-concussive impacts on ocular motor findings. Whether or not a patient is symptomatic may not reflect their neurological health on a biological level, as suggested in recent advanced neuroimaging studies (14; 33; 86; 158; 159). It is important to discern at what point biological recovery is sufficient to guide safe return to sport (i.e. when the patient is no longer in a period of increased cerebral vulnerability). This is particularly relevant in paediatric mTBI cohorts where evidence is mixed and generally lacking. Altogether, these limitations prevent the definitive selection of any ocular biomarkers in mTBI.

## Summary

The variety of ocular motor dysfunction highlights the diffuse and highly integrated brain circuitry of cortical, subcortical, and cerebellar structures which may be vulnerable to damage in mTBI. Accordingly, mTBI’s effect on ocular motility may occur from disruption to these networks, producing errors not readily appreciated on routine clinical assessment. Ocular motor measures such as saccades are readily detected with eye tracking technology, showing increased latencies, decreased accuracy (higher mean position error), and impaired ability to generate self-paced saccades which may be attributed to higher cortical impairment (e.g. cognition or attention). However, as Nij Bijvank and colleagues highlight in their standardized protocol for quantification of saccadic eye movements, test-retest reliability is poor for more complex saccadic tasks even in healthy participants which makes it a poor discriminator on an individual level (134). This has serious implications for its use in mTBI, but also supports a standardized protocol among researchers. Smooth pursuit studies reveal higher lag times, lower tracking accuracy, and difficulty synchronizing to visual targets, depending on patients’ recovery status. This dysfunction also appears greater during higher cognitive loads in mTBI patients, highlighting cognition and attention effects. However, VOR does not reveal similar results with little sensitivity even when measured quantitatively, unless a computer-controlled, rotational chair is used. However, oVEMPs are an emerging area of research which may prove more sensitive to peripheral vestibular injury in mTBI.

Measures of ciliary body function, such as the pupillary light reflex and accommodation, have shown decreased constriction and dilation velocities with altered resting pupil diameter. This may suggest autonomic disruption or damage to both afferent and efferent pathways from biomechanical forces with a currently unknown recovery trajectory. However, this area requires further investigation with no conclusive evidence to date. Both individual and experimental confounding variables (e.g. past medical history, medications, mental state, attention) influence the interpretation of this area. Likewise, accommodative and vergence dysfunction (including recovery) show equally mixed evidence. Importantly, measures of near-vision may serve as a useful biomarker in predicting time off work or delayed return to school. Recovery of a near-vision biomarker could inform a medical decision for return to work.

## Conclusion

mTBI is a global health issue with complexities in diagnosis, prognostication, and management. Currently, the perfect ocular biomarker does not exist. However, studies have shown varying degrees of ocular motor dysfunction in mTBI with emerging evidence suggesting its utility as global index of cognitive dysfunction, rather than primary damage to ocular motor systems. This is highlighted by studies showing greater degrees of dysfunction following higher cognitive loads (42; 74; 77; 114; 163). In the future, eye tracking may prove to be a reliable, portable, and sensitive biomarker for mTBI, but this area is in its infancy. A global metric, or weighted score, involving a combination of ocular motor measures (e.g. vergence indices with smooth pursuit accuracy and complex saccadic task measurements) may prove most sensitive to mTBI. Future directions of research should include a combination of reliable outcome measures for developing practical, rapid, and inexpensive tools. These must be validated using longitudinal cohorts with careful attention to methodology and assessment of diagnostic accuracy on an individual level. Finally, these findings must be translated into user-friendly instruments available to clinicians and allied health professionals.

### Ethics and Conflict of Interest

The author(s) declare(s) that the contents of the article are in agreement with the ethics described in http://biblio.unibe.ch/portale/elibrary/BOP/jemr/ethics.html and that there is no conflict of interest regarding the publication of this paper.

### Acknowledgements

This research was supported by the Health Research Council of New Zealand through the Clinical Research Training Fellowship.
